# Reference Range of Vitamin K Evaluating Indicators in Chinese Childbearing Women

**DOI:** 10.3390/nu15081977

**Published:** 2023-04-19

**Authors:** Shuhui Nie, Lichen Yang, Jie Feng, Jiaxi Lu, Huidi Zhang, Weidong Li, Yichun Hu, Xiaoguang Yang

**Affiliations:** 1 National Institute for Nutrition and Health, Chinese Center for Disease Control and Prevention, Key Laboratory of Trace Element Nutrition, National Health Commission of the People’s Republic of China, Beijing 100050, China; nsh_0120@163.com (S.N.); yanglc@ninh.chinacdc.cn (L.Y.); fengjie@ninh.chinacdc.cn (J.F.); lujx@ninh.chinacdc.cn (J.L.); zhanghuidi1114@126.com (H.Z.); liwd@ninh.chinacdc.cn (W.L.); 2 Chinese Nutrition Society, Beijing 100050, China

**Keywords:** vitamin K, nutritional status, reference range, Chinese childbearing women

## Abstract

Background: Vitamin K is an essential fat-soluble vitamin for the human body and its functions, such as promoting blood coagulation, bone health and preventing atherosclerosis, have attracted increasing attention. However, there is no recognized indicator and corresponding reference range for evaluating vitamin K status of different populations at present. The aim of this study is to establish a reference range for vitamin K evaluating indicators in healthy women of childbearing age in China. Methods: The population sample in this study was from the Chinese Adult Chronic Disease and Nutrition Surveillance (CACDNS) 2015–2017. A total of 631 healthy women of childbearing age (18–49 years) were included using a series of strict inclusion and exclusion criteria. The concentrations of VK1, MK-4 and MK-7 in serum were detected by liquid chromatography–tandem mass spectrometry (LC–MS/MS). The other commonly-reported indicators evaluating vitamin K nutritional status, including undercarboxylated osteocalcin (ucOC), osteocalcin (OC), matrix Gla protein (MGP), desphosphorylated undercaboxylated MGP (dp-ucMGP) and protein induced by vitamin K absence II (PIVKA-II), were measured by enzyme-linked immunosorbent assay (ELISA). The reference range was obtained by calculating the 2.5% to 97.5% interval of the vitamin K evaluating indicators in the reference population. Results: The reference ranges of VK1, MK-4 and MK-7 in serum were 0.21–3.07 ng/mL, 0.02–0.24 ng/mL and 0.12–3.54 ng/mL, respectively. The reference ranges of ucOC, %ucOC, dp-ucMGP and PIVKA-II were 1.09–2.51 ng/mL, 5.80–22.78%, 2.69–5.88 ng/mL and 3.98–8.40 ng/mL, respectively. The cut-off values that can be used to evaluate subclinical vitamin K deficiency were as follows: VK1 < 0.21 ng/mL, MK-7 < 0.12 ng/mL, ucOC > 2.51 ng/mL, %ucOC > 22.78%, dp-ucMGP > 5.88 ng/mL and PIVKA-II > 8.40 ng/mL. Conclusion: The reference range of VK1, MK-4, MK-7 and vitamin K-related indicators for healthy women of childbearing age established in this study could be used to assess the nutritional and health status of this population.

## 1. Introduction

Vitamin K (VK) is an essential fat-soluble vitamin, including phylloquinone (PK or VK1) and menaquinones (VK2 or MK-n) in nature. VK1 is widely found in green vegetables because it is synthesized in the chloroplast of plants and VK2 is found in various meats, dairy products and fermented foods [1,2]. The coagulation function of vitamin K has been well-known after its discovery and other health effects of vitamin K, such as maintaining bone strength, preventing the development of vascular calcification and reducing risk of diabetes [2,3,4], have gradually received increasing attention. A growing number of evidence shows that low vitamin K intake or status is associated with higher risk of bone fracture, cardiovascular disease, chronic kidney disease (CKD) and other diseases [1,5]. Since vitamin K plays a variety of health roles in the human body and its insufficiency or deficiency may affect normal physiological health, there is a great need to assess the nutritional status of VK in different populations.

However, there is no recognized evaluation indicator or relevant reference range for evaluating vitamin K status. Many studies have reported that vitamin K status could be assessed by serum or plasma vitamin K concentration directly [6,7] or by vitamin K-dependent protein (VKDP) levels indirectly, such as undercarboxylated osteocalcin (ucOC), desphosphorylated undercaboxylated matrix Gla protein (dp-ucMGP) and protein induced by vitamin K absence II (PIVKA-II) [8,9,10,11]. The level of ucOC and dp-ucMGP are recognized as markers of vitamin K in bone metabolism and vasculature, respectively [12,13]. The nutritional status of vitamin K in people with different physiological and disease states may also be affected by various factors, such as the prevalent suboptimal vitamin K status in hemodialysis patients [14]. At present, there is no unified reference range for each indicator mentioned above, not to mention the reference ranges for different populations or under different physiological statuses. Therefore, it is necessary to establish reference ranges for vitamin K evaluating indicators to better assess the nutritional status of different populations.

Women of childbearing age are at an important stage in their lives. It has been shown that vitamin K status affects inflammatory status and bone formation in young adult women [15]. If they suffer from malnutrition or vitamin K deficiency, it may have adverse effects on their own health and that of their offspring. Therefore, we selected childbearing women as the population for this study and we also selected a series of more potential and more frequently reported indicators in the literature to establish corresponding reference range, including serum vitamin K, ucOC, osteocalcin (OC), ratio of ucOC to OC (%ucOC), matrix Gla protein (MGP), dp-ucMGP and PIVKA-II. The purpose of this study is to establish reference ranges of vitamin K evaluating indicators based on healthy women of childbearing age in China, so as to accumulate scientific data for better evaluation of vitamin K nutritional status.

## 2. Materials and Methods

### 2.1. Subjects

All the subjects in this study were selected from the biological sample bank of Chinese Adult Chronic Disease and Nutrition Surveillance (CACDNS) 2015–2017. The CACDNS 2015–2017 is a representative survey, covering 31 provincial administrative units in the Chinese mainland. All the samples were selected using the simple random sampling method. Considering the representativeness of the samples, the number of survey points (298 actual survey points) and the age distribution, we selected 2 samples for each age group (18–29 years, 30–39 years, 40–49 years) at each survey site. Initially, 1788 women of childbearing age, between 18 and 49 years, were selected. Then, 631 healthy women with the following indicators within the normal range were finally selected for this study. The inclusion criteria in conjunction with the Chinese clinical reference range were as follows: body mass index (BMI, 18.5–24 kg/m^2^), systolic blood pressure (SBP, 90–140 mmHg), diastolic blood pressure (DBP, 60–89 mmHg), fasting glucose (FG, 3.9–6.1 mmol/L), hemoglobin A1c (HbA1c, 4–6%), hemoglobin (Hb, 115–150 g/L), uric acid (UA, ≤357 μmol/L), total cholesterol (TC, <5.2 mmol/L), triglyceride (TG, <1.7 mmol/L), low-density lipoprotein cholesterol (LDL-C, <3.12 mmol/L) and high-density lipoprotein cholesterol (HDL-C, >1.04 mmol/L). Subjects who had been diagnosed with any of the following diseases—hypertension, diabetes, dyslipidemia, coronary heart disease, stroke, chronic gastrointestinal disease and malignant tumor—were excluded. All the subjects signed the informed consent form. This survey was conducted in accordance with the Declaration of Helsinki and approved by the Ethics Committee of the National Institute of Nutrition and Health, Chinese Center for Disease Control and Prevention (China CDC, No. 201614).

### 2.2. Data Collection and Sample Detection

Basic information, including age, residence and latitude, was obtained through questionnaires, which were completed by the uniformly-trained investigators according to the unified standard. BMI was calculated from height and weight that were measured by unified method and equipment. SBP (mmHg) and DBP (mmHg) were measured using Omron HBP1300 sphygmomanometer. Fasting venous blood samples were centrifuged at 1500× *g* for 15 min, 20–30 min after being collected. All blood samples were frozen at −70 °C for subsequent detection and analysis. The detection of Hb was completed by the local testing agency and the detection of other indicators was uniformly organized by the National Institute of Nutrition and Health, China CDC. Total cholesterol, TG, LDL-C, HDL-C, FG and UA were measured enzymatically using an automated biochemistry analyzer (Hitachi 7600, Tokyo, Japan). HbA1c was determined by high performance liquid chromatography ((HPLC), Waters E2695, Milford, MA, USA).

The concentration of VK1, MK-4 and MK-7 in serum was detected by the liquid chromatography–tandem mass spectrometer (LC–MS/MS). Liquid phase separation was performed on a Kinetex Phenyl-Hexyl chromatographic column under gradient flow of eluent, with mobile phases consisting of water with 0.1% formic acid and methanol with 0.1% formic acid. The gradient elution lasted for 5.5 min. The atmospheric pressure chemical ionization source (APCI) operated in positive ion mode and the scan type was multiple reaction monitoring (MRM). As there is no commercially available serum VK2 quality control product, serum vitamin K concentration assays were performed using a uniform quality control scheme, spiked recovery method. Specifically, we added different amounts of standards to blank serum to achieve low, medium and high concentrations as a set of quality control products (QCs) and performed a set of QCs before and at the end of each analytical batch. Both the intra-assay coefficient of variation (CV) and recovery rate were within the preset acceptable range (Table 1).

The other indicators, including ucOC, OC, MGP, dp-ucMGP and PIVKA-II were measured by enzyme-linked immunosorbent assay ((ELISA), MLBIO Biotechnology, Shanghai, China). The quality of the above 5 indicators was ensured by the use of in-kit quality control products and the use of 5% parallel samples detection. The CVs for ucOC, OC, MGP, dp-ucMGP and PIVKA-II were 3.22%, 3.69%, 3.42%, 3.45% and 3.72%, respectively.

### 2.3. Variables and Statistic Analysis

The types of all the subjects were divided into urban and rural areas according to the economic level [16]. Latitude was marked as north and south along the boundary of the Qinling Mountains–Huaihe River line. The serum concentration of all the indicators described in Section 2.2 was expressed in the form of P50 (P25–P75), since they did not conform to the normal distribution. Kruskal–Wallis test was used for comparison between subgroups. According to the recommendation of International Federation of Clinical Chemistry (IFCC), if the sample size was greater than 120, the reference range could be determined according to the concentration estimates from 2.5th to 97.5th percentiles as the reference samples [17]. All the data were analyzed by SAS 9.4 software (SAS Institute, Cary, NC, USA) and *p* < 0.05 was considered as statistically significant by two-sided tests.

## 3. Results

### 3.1. Basic Information

**Figure 1:**
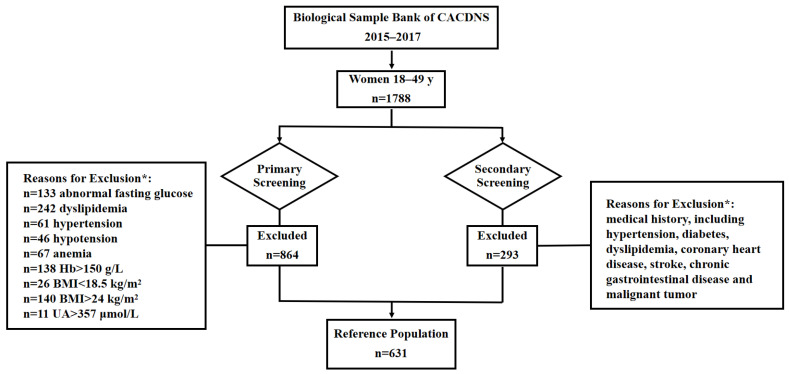
Exclusion schematic to establish reference range for vitamin K evaluating indicators in Chinese women of childbearing age. CACDNS, Chinese Adult Chronic Disease and Nutrition Surveillance; Hb, hemoglobin; BMI, body mass index; UA, uric acid. * Healthy volunteers were obtained by sequential exclusion based on the above criteria.

After screening based on the exclusion criteria, 631 women of childbearing age were included (Figure 1), with a median age of 37.49 years. Among them, 37.56% were from urban areas and 62.44% were from rural areas. 44.37% and 55.63% were from the north and the south, respectively. The basic demographic and clinical data (SBP, DBP, FG, HbA1c, Hb, UA, TC, TG, LDL-C and HDL-C) are summarized and presented in Table 2.

### 3.2. Concentration of Vitamin K in Serum

The median concentration of VK1 in serum was 0.82 ng/mL. MK-4 was detected in only 366 (58.00%) subjects and the concentration was 0.06 (0.03–0.10) ng/mL. The MK-7 concentration was relatively high, 0.55 (0.36–0.82) ng/mL (Table 3). In terms of the overall distribution, the concentration of VK1 showed a significant upward trend with the increase of age (*p* = 0.016) and the subsequent analysis showed that the serum VK1 concentration of women aged 40–49 was higher than that of women aged 18–29. The concentrations of VK1 and MK-4 in the south were higher than those in the north (*p* < 0.05). The concentration of MK-7 in rural population was higher than that in urban population (*p* < 0.001).

### 3.3. Concentration of Vitamin K-Related Indicators in Serum

The median concentrations of ucOC and OC were 1.81 ng/mL and 16.26 ng/mL, respectively, and the ratio of ucOC to OC (%ucOC) was 11.10%. The levels of ucOC, OC and %ucOC were statistically significant in different age groups (*p* < 0.05). The ucOC concentration of northerners was lower than that of southerners (*p* = 0.039). The median concentrations of MGP, dp-ucMGP and PIVKA-II were 1.99 ng/mL, 4.41 ng/mL and 6.26 ng/mL, respectively. No difference was found among different subgroups in terms of MGP, dp-ucMGP and PIVKA-II concentration (Table 4).

### 3.4. Reference Range for Vitamin K Evaluating Indicators

The reference ranges for vitamin K evaluating indicators in Chinese women of childbearing age were established by using concentration data of P2.5 to P97.5 for each indicator (Table 5). The reference range of VK1, MK-4, MK-7, ucOC, %ucOC, dp-ucMGP and PIVKA-II were 0.21–3.07 ng/mL, 0.02–0.24 ng/mL, 0.12–3.54 ng/mL, 1.09–2.51 ng/mL, 5.80–22.78%, 2.69–5.88 ng/mL and 3.98–8.40 ng/mL, respectively.

We also compared the reference ranges we established with the corresponding indicator ranges reported nationally and internationally in healthy subjects. We included all the reports from healthy populations with data on relevant indicators for comparison. We found that only a small amount of literature reported reference ranges for one or some of the indicators mentioned in our study and most of the literature reported only the mean values of some relevant indicators without providing reference ranges (Table 6 and Table 7).

## 4. Discussion

There have been reports on vitamin K levels in populations with different physiological and disease status and some studies have used different indicators to evaluate the vitamin K status. Among then, analysis of the concentration of vitamin K may be considered as a direct method to evaluate the status of vitamin K [6,7] and the VKDPs are indicators of indirect evaluation, in which ucOC and dp-ucMGP are recognized markers of extrahepatic vitamin K status and are, respectively, related to osteoporotic fractures and arterial calcification [2,13]. However, there are too few studies which focus on the reference range and there are still neither recognized indicators nor corresponding reference ranges for evaluating vitamin K status. In this study, we selected 631 healthy Chinese childbearing women to establish reference ranges for a series of indicators to evaluate the nutritional status of vitamin K.

In our study, the mean concentration of VK1 in serum was 1.03 ng/mL, higher than that reported by Klapkova et al. (0.49 ± 0.40 ng/mL) in Europe [31]. The reference range of VK1 for healthy women aged 18–49 years was 0.21–3.07 ng/mL, which is close to the normal range (P5–P95, 0.17–3.05 ng/mL) reported by Fusaro et al. based on healthy adults in Italy [21]; however, both the lower and upper limits are higher than that of the normal range (0.29–2.65 nmol/L, 0.13–1.19 ng/mL) considered by Holden et al. [9]. We suggest that 0.21 ng/mL may be the lower limit of normal VK1 status, which is slightly higher than the cut-off value (0.40 nmol/L, 0.18 ng/mL) reported by Shea et al. in USA [5].

The mean concentration of MK-4 was 0.08 ng/mL, which is close to that (0.07 ng/mL) in healthy Japanese women aged 30–49 years [11]. The reference range of MK-4 was 0.02–0.24 ng/mL and its upper and lower limits are lower than that of the normal range (0.07–2.68 ng/mL) reported in Italy [21]. Because the concentration of MK-4 was not detected in 42% subjects, which has also been reported in other reports [11], and MK-4 concentration in serum is relatively low and can be affected by the conversion of VK1 [32], we do not recommend a cut-off value of MK-4 in this study. The mean concentration of MK-7 was 0.85 ng/mL, much lower than that (4.96 ng/mL) in healthy women aged 30–49 years in Japan, where the major contributor to vitamin K concentration is MK-7 [11]. The difference may be related to dietary habits, in that Japanese people consume natto, a soy product rich in MK-7, more often [2,33]. The reference range of MK-7 was 0.12–3.54 ng/mL, the lower limit of which is lower than the normal range (0.33–4.48 ng/mL) reported in Italy [21]. MK-7 has higher bioavailability and longer half-life than other vitamin K homologues and its health effects on osteoporosis, cardiovascular disease, diabetes and other diseases have gradually gained recognition in recent years [34,35,36]. Therefore, for the sake of health effects, we suggest that the level of MK-7 should be higher than 0.12 ng/mL.

In the condition of vitamin K insufficiency or deficiency, a small amount of ucOC is released from osteoblasts into circulation. Therefore, the concentration of ucOC in serum has been considered as a sensitive marker of vitamin K status in bone [11]. This study showed that the mean ucOC concentration was 1.80 ng/mL, which is lower than that of healthy women reported in Japan (3.59 ng/mL) [11] and Korea (2.02 ng/mL) [23]. In Thailand, Bunyaratavej et al. reported that the median concentration was 2.10 ng/mL in 357 healthy female volunteers aged 20–50 years [12], which is higher than ours (1.81 ng/mL). The reference range of ucOC in our study was 1.09–2.51 ng/mL. Our upper limit is slightly higher than that (2.31 ng/mL) of normal premenopausal level reported by Soontrapa et al. [22] but far lower than that (5.00 ng/mL) in adults over 20 years of age reported by Theuwissen et al. [13]. Considering the negative correlation between ucOC and vitamin K status, we suggest that the upper limit of ucOC concentration in healthy childbearing women should be 2.51 ng/mL.

There are large differences between individuals in terms of OC and it also changes with age. Therefore, the current study concluded that the %ucOC can better reflect the nutritional status of vitamin K. The value of %ucOC is considered to be a sensitive indicator of vitamin K status, especially in bones of non-CKD population [6,9]. This study showed that the reference range of %ucOC was 5.80–22.78%. McKeown et al. found that the median level of %ucOC in plasma was ≤20% when the dietary VK1 intake exceeded the recommended intake AI value in their study population [37]. Holden et al. took %ucOC > 20% as subclinical vitamin K deficiency [9], which is close to the upper limit in our study. Considering that higher %ucOC level is related to lower vitamin K status, we recommend that %ucOC > 22.78% be considered as subclinical vitamin K deficiency.

It has been confirmed that MGP is a potential inhibitor of vascular calcification [38]. Due to different transformation processes, MGP exists in the cycle in various forms, of which dp-ucMGP is considered to be a more reliable indicator of vitamin K status and a better predictor of cardiovascular disease [39]. The median concentration of dp-ucMGP was 4.41 ng/mL, which is close to 399 pmol/L (4.23 ng/mL) in Danish adult women aged 19–49 years [40] but lower than 575 pmol/L (6.10 ng/mL) in healthy women in Netherlands [25]. However, although our value was similar to that of the Danish study, it was not explicitly stated in their study that their participants were a healthy population. The reference range in our study was 2.68–5.88 ng/mL. Griffin et al. obtained that the reference interval of dp-ucMGP was <300–532 pmol/L (3.18–5.64 ng/mL) based on 141 healthy Caucasian adults with median age of 30.0 years recruited with strict inclusion and exclusion criteria, the upper limit of which was generally close to our study [26]. For the level of dp-ucMGP, negatively correlated with vitamin K status, >5.88 ng/mL could be considered as subclinical vitamin K deficiency.

PIVKA-II concentration reflects hypocarboxylated prothrombin and can be used to detect subclinical vitamin K deficiency [41]. There is no consensus on the reference range of PIVKA-II at present. In our study, the concentration and reference range of PIVKA-II were 6.26 (5.24–7.28) ng/mL and 3.98–8.40 ng/mL (7.96–16.80 mAU/mL), respectively. Ko et al. established a reference interval (13.00–37.40 mAU/mL) based on 204 healthy individuals, which is between the Japanese range (11.12–32.01 mAU/mL) and the European range (17.36–50.90 mAU/mL) in the product package insert [27], and the upper limit of these ranges is higher than ours. In addition to indicating subclinical vitamin K deficiency, PIVKA-II can serve as a biomarker of hepatocellular carcinoma (HCC) with cut-off values well above our reference range [30,42]. The results obtained may vary with the selection of different detection assays and kits. Based on our experimental conditions, concentration of PIVKA-II > 8.40 ng/mL can be considered to be at risk for subclinical vitamin K deficiency.

The strength of this study lies in the fact that the sample data were drawn from a nationally representative survey in China. Additionally, the inclusion and exclusion criteria adopted to recruit subjects are relatively strict, taking into account factors that may affect health status as much as possible. In addition, we selected vitamin K evaluating indicators containing direct indicators, such as serum vitamin K concentration, and indirect indicators, such as undercarboxylated VKDP, which can provide a basis for evaluating the nutritional status of vitamin K from different aspects. Finally, the method chose for determining vitamin K concentration was LC–MS/MS, which is a mature quantitative method with fast analysis speed, high sensitivity and accurate quantification [43].

We also admit there are some limitations to this study. Firstly, in order to uniformly detect all indicators and ensure the consistency of detection, we chose ELISA for the detection of ucOC, dp-ucMGP and PIVKA-II. There are different methods to detect these indicators separately, which may lead to bias when comparing the results. Secondly, though 631 healthy women were included in this study to establish the reference range— which is more than the sample size of 120 recommended by IFCC—further validation is still required to determine whether it is appropriate for extrapolation.

## 5. Conclusions

In conclusion, this study, based on 631 healthy childbearing women aged 18–49 years in China, established reference ranges of indicators for evaluating vitamin K nutritional status and proposed partial cut-off value for subclinical vitamin K deficiency. These data can be used to assess the nutritional and health status of this population.

## Figures and Tables

**Table 1:** The result of quality control for VK1, MK-4 and MK-7.

Concentrations	VK1		MK-4		MK-7	
	CV (%)	Recovery Rate (%)	CV (%)	Recovery Rate (%)	CV (%)	Recovery Rate (%)
low concentration	4.38	99.35	5.50	99.60	8.93	100.72
medium concentration	4.72	101.43	4.10	105.27	9.11	102.96
high concentration	5.53	95.99	4.13	100.59	7.45	102.56

**Table 2:** Basic characteristics of the study population.

Characteristic	P50 (P25–P75)	Characteristic	P50 (P25–P75)
Age (years)	37.49 (29.34–44.12)	Hb (g/L)	138.27 (132.09–144.22)
BMI (kg/m^2^)	21.50 (20.19–22.73)	UA (μmol/L)	236.40 (201.00–269.60)
SBP (mmHg)	115.67 (109.00–122.33)	TC (mmol/L)	4.19 (3.77–4.60)
DBP (mmHg)	71.67 (67.00–76.67)	TG (mmol/L)	0.72 (0.56–0.95)
FG (mmol/L)	4.92 (4.61–5.21)	LDL-C (mmol/L)	2.37 (2.04–2.70)
HbA1c (%)	4.70 (4.40–5.10)	HDL-C (mmol/L)	1.40 (1.25–1.56)

BMI, body mass index; SBP, systolic blood pressure; DBP, diastolic blood pressure; FG, fasting glucose; HbA1c, hemoglobin A1c; Hb, hemoglobin; UA: uric acid; TC, total cholesterol; TG, triglycerides; LDL-C, low-density lipoprotein cholesterol; HDL-C, high density lipoprotein cholesterol.

**Table 3:** Concentration of vitamin K in serum.

Characteristic	N (%)	VK1		MK-4		MK-7	
		ng/mL	*p*	ng/mL	*p*	ng/mL	*p*
Total	631	0.82 (0.54–1.31)		0.06 (0.03–0.10)		0.55 (0.36–0.82)	
Age (years)			0.016		0.779		0.023
18–29	171 (27.10)	0.73 (0.46–1.20) ^b^		0.06 (0.03–0.12)		0.51 (0.38–0.76) ^ab^	
30–39	200 (31.70)	0.80 (0.56–1.33) ^ab^		0.05 (0.03–0.09)		0.50 (0.33–0.78) ^b^	
40–49	260 (41.20)	0.88 (0.59–1.43) ^a^		0.06 (0.03–0.10)		0.61 (0.39–0.88) ^a^	
Residence			0.846		0.792		<0.001
Urban	237 (37.56)	0.81 (0.58–1.31)		0.06 (0.03–0.10)		0.49 (0.35–0.73)	
Rural	394 (62.44)	0.83 (0.53–1.31)		0.05 (0.03–0.10)		0.59 (0.39–0.93)	
Latitude			0.047		<0.001		0.053
North	280 (44.37)	0.77 (0.52–1.27)		0.04 (0.02–0.10)		0.51 (0.36–0.74)	
South	351 (55.63)	0.85 (0.57–1.41)		0.06 (0.04–0.11)		0.57 (0.37–0.91)	

Notes: MK-4 was detected in 366 subjects. ^a,b^ statistical difference between groups, *p* < 0.05.

**Table 4:** Concentration of vitamin K-related indicators in serum.

Characteristic	N (%)	ucOC (ng/mL)	OC (ng/mL)	%ucOC (%)	MGP (ng/mL)	dp-ucMGP (ng/mL)	PIVKA-II (ng/mL)
Total	631	1.81 (1.50–2.10)	16.26 (12.26–20.43)	11.10 (8.46–14.77)	1.99 (1.64–2.39)	4.41 (3.73–4.98)	6.26 (5.24–7.28)
Age (years)							
18–29	171 (27.10)	1.71 (1.44–2.02) ^a^	16.37 (12.26–20.07) ^b^	10.70 (8.26–14.13)	1.92 (1.67–2.39)	4.41 (3.69–4.93)	6.18 (5.24–7.20)
30–39	200 (31.70)	1.88 (1.51–2.13)	17.85 (13.35–21.52) ^a^	10.61 (8.07–14.20)	1.96 (1.61–2.43)	4.33 (3.61–4.99)	6.12 (5.10–7.31)
40–49	260 (41.20)	1.84 (1.54–2.11)	15.40 (11.43–20.12) ^c^	12.09 (9.04–15.53) ^a^	2.05 (1.66–2.35)	4.40 (3.79–5.00)	6.36 (5.30–7.29)
Residence							
Urban	237 (37.56)	1.78 (1.50–2.05)	16.05 (12.50–20.79)	11.00 (8.07–14.77)	1.95 (1.66–2.40)	4.37 (3.73–5.00)	6.15 (5.26–7.29)
Rural	394 (62.44)	1.82 (1.50–2.11)	16.39 (12.14–20.27)	11.16 (8.62–14.77)	2.01 (1.63–2.38)	4.42 (3.72–4.96)	6.31 (5.24–7.27)
Latitude							
North	280 (44.37)	1.78 (1.46–2.06)	16.06 (12.61–20.19)	10.98 (8.39–14.24)	2.03 (1.67–2.40)	4.29 (3.76–4.98)	6.12 (5.28–7.19)
South	351 (55.63)	1.82 (1.54–2.13) ^a^	16.38 (11.97–21.01)	11.29 (8.49–15.17)	1.97 (1.59–2.36)	4.46 (3.69–4.99)	6.30 (5.19–7.32)

Notes: To convert dp-ucMGP from ng/mL to pmol/L, multiply by 94.299 [18]. ^a,b,c^ statistical difference between groups, *p* < 0.05.

**Table 5:** Reference range for vitamin K evaluating indicators (P2.5–P97.5).

Characteristic	N (%)	VK1 (ng/mL)	MK-4 (ng/mL)	MK-7 (ng/mL)	ucOC (ng/mL)	%ucOC (%)	dp-ucMGP (ng/mL)	PIVKA-II (ng/mL)
Total	631	0.21–3.07	0.02–0.24	0.12–3.54	1.09–2.51	5.80–22.78	2.69–5.88	3.98–8.40
Age (years)								
18–29	171 (27.10)	0.20–2.95	0.02–0.25	0.13–4.32	1.06–2.47	5.94–22.79	2.68–5.87	3.85–8.21
30–39	200 (31.70)	0.21–3.10	0.01–0.28	0.08–3.56	1.18–2.49	5.59–23.21	2.59–5.95	4.11–8.62
40–49	260 (41.20)	0.21–3.15	0.02–0.24	0.13–3.55	1.09–2.54	6.05–22.65	2.76–5.77	3.94–8.40
Residence								
Urban	237 (37.56)	0.25–3.07	0.02–0.27	0.08–3.55	1.10–2.53	5.76–23.14	2.68–5.76	4.00–8.40
Rural	394 (62.44)	0.20–3.10	0.02–0.24	0.13–3.65	1.09–2.49	5.89–22.28	2.74–5.92	3.92–8.47
Latitude								
North	280 (44.37)	0.21–2.62	0.02–0.19	0.15–3.13	1.07–2.49	5.59–21.90	2.68–5.76	4.00–8.47
South	351 (55.63)	0.21–3.41	0.02–0.30	0.09–3.68	1.11–2.53	5.99–23.02	2.72–5.92	3.95–8.40

Notes: MK-4 was detected in 366 subjects. To convert dp-ucMGP from ng/mL to pmol/L, multiply by 94.299 [18].

**Table 6:** Reference range or concentration for vitamin K reported in literature.

Authors (Years of Publication)	Country	Population	Mean Age (Years)	N	VK1 (ng/mL)	MK-4 (ng/mL)	MK-7 (ng/mL)	Reference
this study	China	healthy women	36.95	631	0.21–3.07	0–0.22	0–3.25	
Sadowski et al. (1989)	USA	healthy adults, aged 20–49 years	33.0	131	0.11–1.15 ^#^	-	-	[19]
		healthy women	33.0	77	0.10–1.09 ^#^	-	-	
		healthy adults, aged 20–92 years	-	326	0.13–1.19 ^#^	-	-	
Tsugawa et al. (2006)	Japan	healthy women, aged 30–49 years	45.4	52	0.24–5.60	0.07 ± 0.14	4.96 ± 6.93	[11]
		healthy women, aged 50–69 years	59.6	208	0.13–8.83	0.10 ± 0.19	8.42 ± 11.44	
		healthy women, ≥70 years	74.9	136	0.19–6.67	0.09 ± 0.15	4.21 ± 6.81	
Sogabe et al. (2007)	Japan	healthy men	22.6	60	0.56 ± 0.34	0.07 ± 0.05	6.97 ± 13.30	[20]
Fusaro et al. (2012)	Italy	healthy adults	56.8	62	0.17–3.05 ^#^	0.07–2.68 ^#^	0.33–4.48 ^#^	[21]

Notes: MK-4 was detected in 366 subjects. ^#^ Normal range or reference interval.

**Table 7:** Reference range or concentration for vitamin K-related indicators reported in literature.

Authors (Years of Publication)	Country	Population	Mean Age (years)	N	ucOC (ng/mL)	%ucOC (%)	dp-ucMGP (ng/mL)	PIVKA-II (ng/mL)	Reference
this study	China	healthy women	36.95	631	1.09–2.51	5.80–22.78	2.69–5.88	3.98–8.40	
Bunyaratavej et al. (2005)	Thailand	healthy women, aged 20–50 years	38.5	357	2.10 ± 2.02	-	-	-	[12]
Soontrapa et al. (2005)	Thailand	healthy women, aged 20–50 years	38.5	357	1.89–2.31 ^#^	-	-	-	[22]
Tsugawa et al. (2006)	Japan	healthy women, aged 30–49 years	45.4	52	3.59 ± 2.17	26.00–82.00	-	-	[11]
Kim et al. (2010)	Korea	healthy women	47.8	337	2.02 ± 1.58	-	-	-	[23]
Theuwissen et al. (2014)	Netherlands	healthy adults over 20 years	-	<896	1.50–5.00	-	-	-	[13]
		children	<20	<896	3.40–96.90	-	-	-	
Cranenburg et al. (2010)	Netherlands	healthy adults, aged 20–85 years	-	75	-	-	4.74 ± 1.99	-	[24]
Dalmeijer et al. (2013)	Netherlands	healthy women	64.9	100	-	-	1.08–30.24	-	[25]
Griffin et al. (2019)	Ireland	healthy adults	-	141	-	-	3.17–5.64 ^#^	-	[26]
Ko et al. (2018)	Korea	healthy subjects	-	204	-	-	-	6.50–18.70 ^#^	[27]
Yan et al. (2018)	China	healthy Han women	>18	381				5.98–19.57 ^#^	[28]
Ryu et al. (2019)	Korea	healthy subjects	-	120	-	-	-	6.00–23.50	[29]
Feng et al. (2021)	China	healthy subjects	-	153	-	-	-	8.83–13.27	[30]

Notes: To convert dp-ucMGP from ng/mL to pmol/L, multiply by 94.299 [18]. ^#^ Normal range or reference interval.

## Data Availability

The data presented in this study are available on request from the corresponding author. The data are not publicly available due to our laboratory’s policies.
